# Genetic engineering of pigs for xenotransplantation to overcome immune rejection and physiological incompatibilities: The first clinical steps

**DOI:** 10.3389/fimmu.2022.1031185

**Published:** 2022-12-06

**Authors:** Tiantian Lei, Lin Chen, Kejing Wang, Suya Du, Carmen Gonelle-Gispert, Yi Wang, Leo H. Buhler

**Affiliations:** ^1^ Department of Pharmacy, Women and Children’s Hospital of Chongqing Medical University, Chongqing Health Center for Women and Children, Chongqing, China; ^2^ Department of Clinical Pharmacy, Sichuan Cancer Hospital and Institute, Sichuan Cancer Center, School of Medicine, University of Electronic Science and Technology of China, Sichuan, China; ^3^ Faculty of Science and Medicine, University of Fribourg, Fribourg, Switzerland; ^4^ Department of Critical Care Medicine, Sichuan Academy of Medical Science and Sichuan Provincial People’s Hospital, University of Electronic Science and Technology of China, Chengdu, China

**Keywords:** genetically modified pigs, immunological rejection, innate and adaptive immune responses, coagulation dysfunction, systemic inflammation, pig-to-nonhuman primate xenotransplantation, clinical trials of xenotransplantation

## Abstract

Xenotransplantation has the potential to solve the shortfall of human organ donors. Genetically modified pigs have been considered as potential animal donors for human xenotransplantation and have been widely used in preclinical research. The genetic modifications aim to prevent the major species-specific barriers, which include humoral and cellular immune responses, and physiological incompatibilities such as complement and coagulation dysfunctions. Genetically modified pigs can be created by deleting several pig genes related to the synthesis of various pig specific antigens or by inserting human complement‐ and coagulation‐regulatory transgenes. Finally, in order to reduce the risk of infection, genes related to porcine endogenous retroviruses can be knocked down. In this review, we focus on genetically modified pigs and comprehensively summarize the immunological mechanism of xenograft rejection and recent progress in preclinical and clinical studies. Overall, both genetically engineered pig-based xenografts and technological breakthroughs in the biomedical field provide a promising foundation for pig-to-human xenotransplantation in the future.

## Introduction

Transplantation is a challenging and complex area of modern medicine and is an effective approach to managing patients with organ failure. According to global statistics, more than 106,795 candidates are on the United States’ transplant waiting list (https://www.organdonor.gov/[accessed Jan. 3, 2022]), and approximately 300,000 people are on China’s transplant waiting list annually (1). However, only 129,861 transplantation operations (≤ 10% of global needs) were performed globally in 2020 (http://www.transplant-observatory.org [accessed Jan. 3, 2022]). The rising incidence of vital organ failure, combined with an insufficient availability of organs particularly from deceased donors, has generated an increasing gap between organ availability and need, resulting in extremely long waiting times for patients on waiting lists.

Xenotransplantation, which aims to substitute human failing organs with animal organs, could efficiently solve various organ shortages. Pigs (*Sus scrofa domesticus*) are currently considered the ideal donor animals for human xenotransplantation (2). Despite similarities between pig and human organs regarding size and physiology, major obstacles to clinical application are due to the immune rejection between species, several physiological incompatibilities and the risk of xenogeneic infections or xenozoonoses. Genetic modifications of pigs have be proposed to overcome most of these hurdles. Recent advances in gene-editing systems, including zinc finger nucleases, transcription activator-like effector nucleases, and the clustered regularly interspaced short palindromic repeats (CRISPR)/CRISPR-associated protein (CRISPR-Cas) system, allow for rapid insertion or deletion of specific sites by gene editors that reduce the work and shorten the time necessary to create genetically engineered pigs.

The immune responses for xenotransplants are significantly stronger when compared with allotransplants (**Tables 1**, **2**). Thus, immunological rejection, including hyperacute xenograft rejection (HAR), acute humoral xenograft rejection (AHXR), acute immune cellular rejection, and other barriers associated with xenotransplantation, could be alleviated through more advanced strategies for the genetic modification of pigs. In this review, we detail the adverse results and underlying mechanisms caused by the recipient immune response and immune-related abnormalities in xenotransplantation, focusing on the application of existing genetically modified pigs in solving these obstacles (**Table 2**).

**Table 1:** Obstacles and relevant strategies in human allotransplantation.

Obstacle	Onset	Mechanism	Strategy	Reference
Hyperacute Transplant Rejection (Type II Hypersensitivity)	Minutes to Hours	Naturally occurring or preformed circulating antibody in the serum of the recipient reacts with donor cells (particularly the endothelium of blood vessel walls).	Stringent antigenic matching is performed between host and donor.	(3)
Acute Transplant Rejection (Type IV Hypersensitivity)	Weeks to Months	1. Increased expression of HLA class I and II antigens in inflamed grafts 2. Infiltration of effector cells responsible for the damage of rejection including T cells, macrophages, NK cells, B cells, and etc.	1. Immunosuppressive drugs (eg. azathioprine, corticosteroids). 2. Matching MHC protein alleles between recipient and donor.	(4, 5)
Chronic Transplant Rejection (Type III and IV hypersensitivity)	Months to Years	The recipient T cells become alloreactive to recognize major histocompatibility complex (MHC) antigens on the donated organ, and promote local immune and inflammatory.	1. The treatment of complications induced by organ rejection depends on the type of injury and underlying etiology. 2. Tolerance induction, including mesenchymal stem cell (MSC), regulatory T cell (Treg) or CAR Treg therapy, and thymic transplantation.	(6–10)
Chronic Graft vs. Host Disease (Type IV Hypersensitivity)		Donor T Cells in the graft proliferate and attack the recipient’s tissue (most commonly seen in bone marrow transplantation, liver transplantation, and blood transfusion)	1. Enduring immunosuppressive therapy (eg. post-transplant cyclophosphamide, anti-thymocyte globulin). 2. Tolerance induction.	(9, 11, 12)

**Table 2:** Obstacles and relevant genetic modifications in pig-to-primate xenotransplantation.

Obstacle		Target	Full name	Function	Reference
**Hyperacute rejection**		hCD46	Transgenic human membrane cofactor protein	Inactivating of C3, C5, and membrane attack complex (MAC)	(13–16)
		hCD55	Transgenic human decay-accelerating factor		
		hCD59	Transgenic human membrane inhibitor of reactive lysis		
		GGTA1^-/-^	α-1,3-galactosyltransferase knockout	Deleting the xenoantigen galactose-α-1,3-galactose (α- Gal)	(17, 18)
**Acute humoral xenograft rejection**		CMAH^-/-^	Cytidine monophosphate-N-acetylneuraminic acid hydroxylase knockout	Deleting the xenoantigen N-glycolylneuraminic acid (Neu5Gc) and xenoantigen DBA-reactive glycans (Sda)	(19)
		β4GalNT2^-/-^	β-1,4N-acetylgalactosaminyltransferase 2 knockout		(20)
**Innate cellular xenograft rejection**	Macrophages	hCD47	Transgenic human integrin-associated protein	Regulating macrophage- mediated xenograft rejection	(21)
		hCD200	Transgenic human OX-2 membrane glycoprotein		(22)
		hα2,6-ST	Transgenic human α-2,6-sialyltransferase		(23)
	NK cells	hHLA-E	Transgenic human leukocyte antigen-E	Inhibiting NK cell-mediated cytotoxicity	(24)
		hB2M	Transgenic β-2-microglobulin gene		(24)
		hHLA-G1^-/-^	Transgenic human leukocyte antigen-G1 knockout		(25)
		CIITA^-/-^	Class II transactivator gene knockout		(26)
		B2M^-/-^	β-2-microglobulin gene knockout		(26)
	Innate-like T cells	hFasL	Transgenic human Fas ligand	Regulating xenograft rejection of NK T cell and γδ T cell	(27)
	Neutrophils	hCD31	Transgenic human platelet endothelial cell adhesion molecule-1	Inhibiting NETosis	(28)
	Dendritic cells	hTRAIL	Transgenic human TNF-related apoptosis-inducing ligand	Inhibiting immune effect of pig dendritic cells to human T cell	(29)
**Adaptive cellular xenograft rejection**	T cells	hCIITA-DN	Transgenic human class II transactivator dominant negative	Reducing swine leukocyte antigen class II (SLA-II)	(30)
		*B2M* ^-/-^	β-2-microglobulin gene knockout	Reducing swine leukocyte antigen class I (SLA-I)	(26)
		pCTLA4-Ig	An expression vector containing the extracellular coding region of porcine CTLA4 fused to the hinge and CH2/CH3 regions of human IgG1	Inhibiting CD80/CD86-CD28 axis	(31)
		hCTLA4-Ig	Transgenic human cytotoxic T-lymphocyte-associated protein 4-immunoglobulin	Inhibiting CD80/CD86-CD28 axis	(32)
		hLEA29Y	Transgenic human variant of CTLA4-Ig	Inhibiting CD80/CD86-CD28 axis	(33)
		hPD-L1	Transgenic human programmed cell death ligand 1	Inhibiting PD-1-PD-L1 axis	(34)
**Coagulation disorder**		hTBM	Transgenic human thrombomodulin	Targeting molecular incompatibilities, and correcting imbalance between procoagulant and anticoagulant activity	(35)
		hTFPI	Transgenic human tissue factor pathway inhibitor		(36)
		hCD39	Transgenic human Ectonucleoside triphosphate diphosphohydrolase		(37, 38)
		hEPCR	Transgenic human endothelial protein C receptor		(35, 36)
		h*pVWF	Replace pig von Willebrand Factor gene region with human cDNA orthologs		(39)
		ASGR1^-/-^	Asialoglycoprotein receptor 1 knockout		(40, 41)
**Systemic inflammation**		hHO-1	Transgenic human heme oxygenase-1	Inhibiting inflammation and apoptosis	(42)
		hA20	Transgenic human TNF Alpha Induced Protein 3 (TNFAIP3)		(42)
		shTNFRI-Fc	Transgenic soluble human tumor necrosis factor- α receptor inhibitor-Fc		(43)
**Cross-species infection**		PERV*^-/-^*	Porcine endogenous retrovirus knockout	Elimination of the cross-species transmission risk of PERVs	(44)

### Circumventing HAR *via* the expression of human complement-regulating proteins and the knockout of pig alpha-1,3-galactosyltransferase (GGTA1^-/-^)

**Figure 1:**
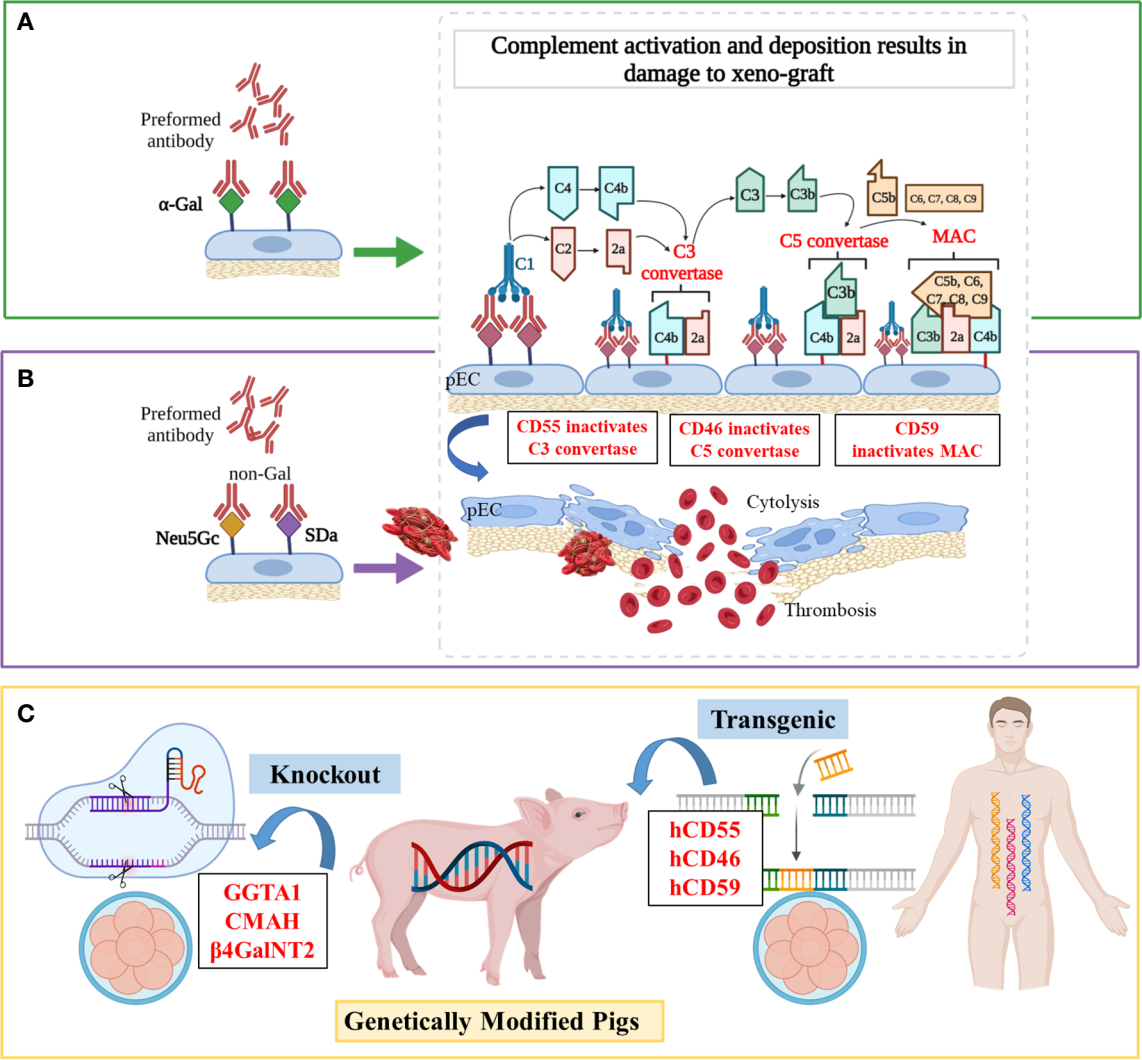
Mechanisms of antibody-mediated xenograft rejection and related genetic targets **(A)** Hyperacute rejection (HAR). In the classical complement activation cascade, the Fc regions of antibodies contact C1q, causing C1r and then C1s to autoactivate. Afterward, C4 and C2 are cleaved by activated C1s, resulting in the production of C4b2a (also known as C3 convertase). C3 convertase then cleaves C3 into C3a and C3b, with C3b binding to C4b2a to generate C4b2a3b (also known as C5 convertase). C5 convertase cleaves C5 into C5a and C5b, and then C5b is attached to C6, C7, C8, and multiple molecules of C9 to create the membrane attack complex (MAC), which ultimately causes cytolysis. **(B)** Acute humoral xenograft rejection (AHXR). AHXR can be induced by low levels of natural and elicited xenoreactive antibodies directed at non-α-Gal antigens (predominantly anti-Neu5Gc and -SDa), which also lead to complement activation *via* the classical pathway. The histopathology of AHXR is characterized by progressive destruction of the microvasculature (glomeruli and peritubular capillaries) and formation of fibrin-platelet thrombi. **(C)** Gene modifications. To prevent HAR, *CD59*, *CD55*, *hCD46* and *GGTA1* were modified. To shun AHXR, *CMAH^-/-^* and *β4GalNT2^-/-^* knockout pigs were produced. Notably, these target genes were not edited individually, but in combination to obtained better tolerized pig xenograft.

HAR occurs when a “wild-type” porcine xenograft is transplanted into primates and is quickly attacked by the recipient’s immune system. The activation of the complement (**Figure 1**) plays a pivotal part in this process, which causes irreversible damage to the donor organ or even death within minutes (45). Antibody-mediated complement activation is completed within minutes and induces graft dysfunction and disruption, the pathological features of which are endothelial edema, interstitial hemorrhage, and microvascular thrombosis (46).

## Complement targeting

The pigs also havec omplement-regulating proteins (pCRPs) but these are not able to protect the endothelial cells of pigs from complement-mediated injury due to molecular incompatibilities between the donor’s and host’s species. Since the 1990s, researchers have introduced transgenes encoding hCRPs into pigs, such as the decay‐acceleration factor (DAF, also known as CD55) (47), membrane cofactor protein (MCP, also known as CD46) (48), and membrane inhibitor of reactive lysis (MIRL, also known as CD59) (13). For example, a *hCD46* transgene alleviated hyperacute kidney graft rejection in non-immunosuppressed baboons by controlling both classical and alternative pathway complement activation (median > 50 h survival, n = 9), and postponed the time before appearance of endothelial swelling, polymorph granulocytes adherence, and lymphocyte infiltration in transgenic kidneys at least until day 3 post-transplant, while baboons receiving wilde-type pig kidneys survived a median of 3.5 h (n = 7) (14). Also, compared with wild-type hearts that survived for 20-80 min, *hCD59*/*hCD55* transgenic pig hearts survived and functioned for 85–130 h in baboons (n = 4) by improved protection from HAR, although IgM deposition was similarly visible both in wild-type and transgenic hearts (15). Recently, Martinez-Alarcon et al. collected organs and tissues from five *hCD55* transgenic commercial Landrace–Large White pigs (*Sus scrofa*) to assess their ability to overcome HAR by classical complement pathway hemolysis assays (16). They observed that the specimens with higher *hCD55* mRNA expression (heart > liver > lung > intestine) performed better in terms of showing lower cytolysis and hemolysis *in vitro*, which enabled researchers to accurately predict the protection level from xenorejection for specific organs.

## Strategies combining GGTA1 knockout and hCRP transgenic pigs

The α,3-galactosyltransferase (α,3GT or GGTA1) is an enzyme present in most mammals except man, apes, and Old World monkeys (49). Preformed xenoreactive antibodies from primate serum recognize and bind the xenoantigen galactose-α-1,3-galactose (α-Gal) on porcine endothelium, which causes the formation of a membrane attack complex and cytolysis *via* complement activation cascade (**Figure 1A**) (50–52). Initial studies of organ transplantation from *GGTA1* knockout (*GGTA1^-/-^*) pigs in baboons showed protection from HAR, with significantly prolonged survival of pig grafts (17, 18, 53, 54).

Different studies validated that combining *GGTA1^-/-^* and hCRP expression is much more effective in preventing early graft failure of a pig organ transplant (55, 56) (**Figure 1C**). Further, Mohiuddin et al. transplanted *GGTA1^-/-^*/*hCD46* hearts into baboons and used anti-CD154 mAb-based immunosuppression to extend heterotopic cardiac xenograft survival to 236 days (57). Despite avoiding HAR, this xenograft developed thrombotic microangiopathy, and coagulation dysregulation was the greatest hurdle to reaching extended survival rates. Similarly, Iwase et al. found that thrombocytopenia and fibrinogen reduction occurred within 21 days in *GGTA1^-/-^*/*hCD46*/*hCD55* hearts, indicating the emergence of thrombotic microangiopathy (58). After immunosuppression therapy including anti-thymocyte globulin (ATG) with anti-CD154 mAb, the recipient baboon survived for 33 days and died from delayed xenograft rejection (DXR). In brief, the expression of hCRPs and the knockout of pig *GGTA1* confers longer survival owing to preferable protection from HAR.

## Shunning AHXR by deleting the genes CMAH and β4GalNT2

Although the histopathologic features of AHXR are similar to those of HAR, AHXR occurs within a few days or weeks, and the accompanying vascular antibody and complement deposition is more variable (59). The non-Gal xeno-antigens can bind to antibodies on endothelial cell surfaces of hosts, which causes complement activation, endothelial activation, cytotoxicity mediated by Natural Killer (NK) cells and macrophages, and other complications, all eventually leading to AHXR (60).

## Removing Neu5Gc and SDa non-Gal antigens

Non-Gal antigens include sialic acid N-glycolylneuraminic acid (Neu5Gc), synthetized by the product of the cytidine monophospho-N-acetylneuraminic acid hydroxylase (*CMAH*) gene, and a carbohydrate antigen (SDa), synthetized by the product of the porcine β1,4-N-acetylgalactosaminyltransferase 2 (*β4GalNT2*) gene (**Figure 1B**) (61, 62). Owing to a divergent evolution approximately 3 million years ago, which was accompanied by the loss of the CMAH hydroxylase activity required to convert Neu5Ac into Neu5Gc, humans do not produce Neu5Gc (63). However, *CMAH* is functional in all mammals (*e.g.*, pigs and Old World monkeys) except humans and New World monkeys (64). As the great majority of humans were exposed to dietary Neu5Gc since childhood, natural anti-Neu5Gc antibodies (with a preponderance of IgG) have been found in approximately 80% of humans at similar levels (65, 66). Although the SDa antigen is not antigenic for all humans, the sera of 90% of humans showed antibodies that are directed against antigens produced by β4GalNT2 activity in pigs (67).

Adams et al. transplanted kidneys from *GGTA1^-/-^*/*β4GalNT2* ^-/-^ pigs into Rhesus monkeys (n = 6) who had received an immunosuppressive regimen consisting of anti-CD4 and anti-CD8 T-cell depletion, anti-CD154, mycophenolic acid, and steroids (20). Three kidney grafts, therein, were rejected early by IgM antibody-mediated rejection at 5 and 6 days after transplantation, and presented with interstitial hemorrhage. Moreover, one 435-day kidney graft was rejected by IgG antibody-mediated rejection, characterized by advanced glomerulopathy rather than significant proteinuria (20).

To further characterize glycan-based species incompatibilities, Estrada et al. created *GGTA1^-/-^*/*CMAH^-/-^*/*β4GalNT2^-/-^* pigs and found that the peripheral blood mononuclear cells (PBMCs) from these pigs exhibited reduced human IgM and IgG reactivity compared to cells lacking GGTA1 and CMAH (19). Because Neu5Gc is expressed on porcine aortic and pulmonary valves and pericardium, removing this antigen through *CMAH* knockout could reduce antibody response and cardiac valve calcification in pig-to-human heart tissue xenotransplantation (68, 69). In addition to the binding of IgM/IgG antibodies from primates to pigs, Li et al. investigated the binding of natural preformed and elicited IgE/IgA antibodies from primates against the erythrocytes from *GGTA1^-/-^*/*CMAH^-/-^*/*β4GalNT2^-/-^* pigs and found IgE/IgA deposition in rejected pig xenografts (**Figure 1C**) (70). Moreover, Martens et al. found that in renal transplant-waitlisted patients, serum IgM/IgG reactivity against PBMCs from *GGTA1^-/-^*/*CMAH^-/-^*/*β4GalNT2* ^-/-^ pigs was decreased compared to that against PBMCs from other donor pigs (71).

## Cellular xenograft rejection in pig-to-nonhuman primate transplantation and related gene modifications

Unlike HAR and AHXR, cellular xenograft rejection is relevant to both whole organ grafts and cellular grafts, and rejection occurs within days to weeks after xenotransplantation (72). As the HAR/AHXR-mediated complement response occurs more rapidly and strongly than cellular response, research on the role and pathological features of cellular xenograft rejection in xenotransplantation is more difficult to identify. Yet, all mechanistic studies are required for the eventual success of xenotransplants, and no immune mechanisms should be less explored.

## Avoiding innate cellular xenograft rejection *via* genetic modifications

The phagocytic cells (monocytes/macrophages and neutrophils), natural killer (NK) cells, and cells producing inflammatory mediators (basophils, eosinophils, and mast cells) are engaged in the innate cellular response (73, 74). If the HAR caused by antibody-mediated complement activation is avoided, the innate cellular response contributes to the development of a delayed form of rejection described as acute humoral xenograft rejection, acute vascular rejection, or DXR (75).

## Macrophages

Abundant cellular infiltrate, composed of polymorphonuclear leukocytes and CD68^+^ macrophages, was observed in *GGTA1^-/-^* pig kidneys (n = 7, pig-to-baboon xenotransplantation) during the early post-transplantation period, which suggests the involvement of macrophages in innate cellular xenograft rejection (76). Activated macrophages may exert direct toxic effects on xenografts through production of proinflammatory cytokines, such as interleukins, tumor necrosis factor alpha (TNF-α), and interferon gamma (IFN-γ) (77). Macrophages mediate robust rejection of donor hematopoietic cells in a variety of xenogeneic settings, through the combined effects of CD47-signal regulatory protein α (SIRP-α) inhibitory receptor signaling, CD200-CD200R signaling, IFN-γ, and danger associated molecular pattern (DAMP)-toll-like receptor (TLR) signaling from damaged porcine cells (78).

**Figure 2:**
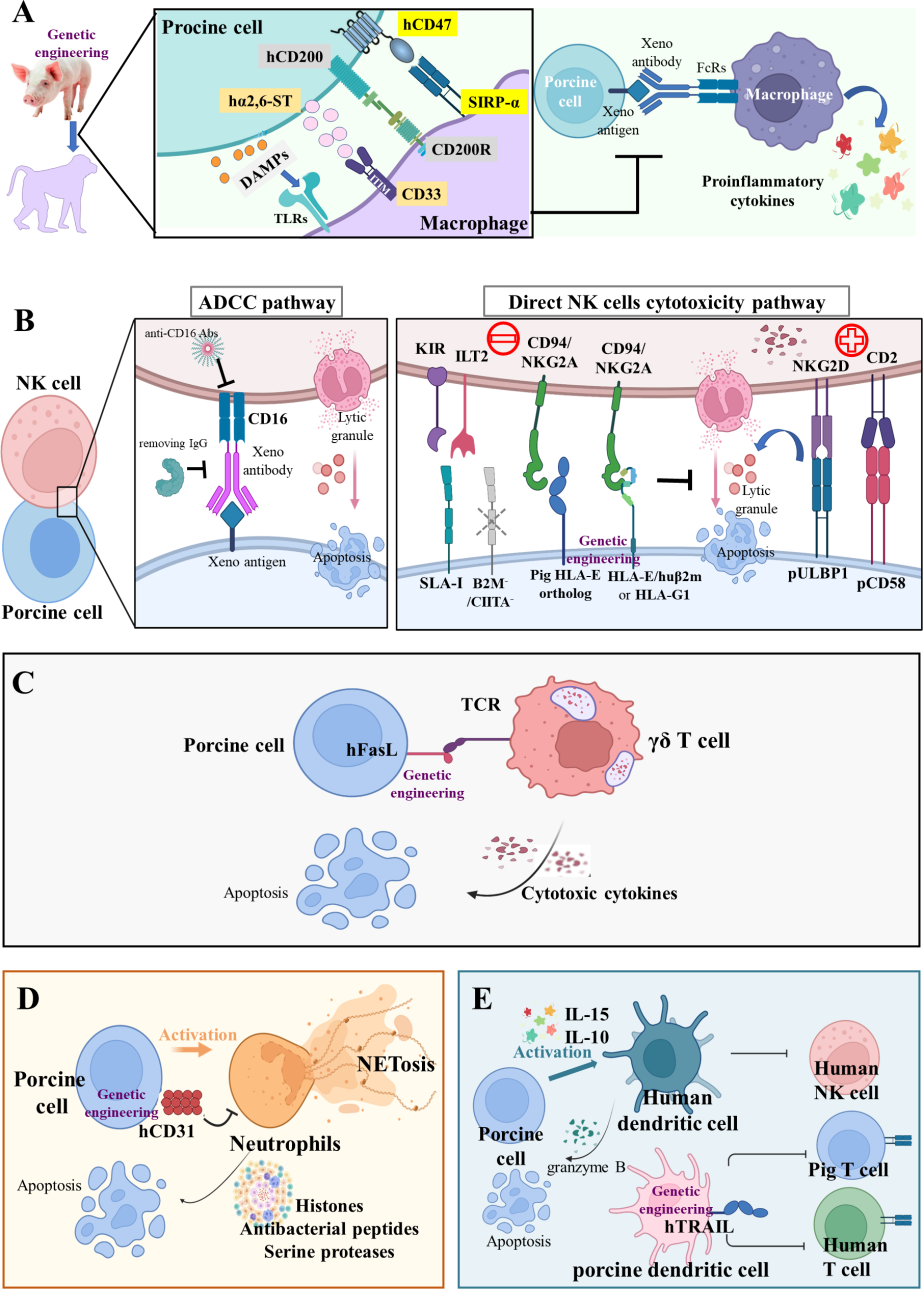
Innate cellular xenograft rejection **(A)** Macrophage-mediated rejection. Macrophages mediate robust rejection of donor hematopoietic cells in a variety of xenogeneic settings; this potent xenoreactivity results from the combined effects of CD47-SIRPα inhibitory receptor signaling, CD200-CD200R signaling, CD33-Siglecs signaling, and DAMPs from damaged porcine cells. The relevant genetic modifications of macrophage-mediated rejection are *hCD47*, *hCD200*, and *hα2,6-ST* transgenes. **(B)** NK-cell-mediated rejection. NK cells may induce pig endothelium lysis *via* two main immune mechanisms of antibody-dependent cytotoxicity (ADCC) and direct NK cell cytotoxicity. During ADCC, the NK cells recognize and bind to the Fc region of antibody deposits through their CD16 and then release cytotoxic granules. In the direct NK cells’ cytotoxicity pathway, the inhibitory receptors on human NK cells, including KIR, ILT2, and CD94/NKG2A, poorly recognize the SLA-I and pig HLA-E ortholog, thus disabling inhibitory signals for NK cell activation. In addition, the upregulation of pULBP1-NKG2D and pCD58-CD2 activating signals could lead to direct NK cell cytotoxicity. The relevant genetic modifications of NK-mediated rejection are *HLA-E/hB2M* transgene and *B2M* ^-/-^/*CIITA* ^-/-^ knockout. **(C)** Innate-like T cell-mediated rejection. The γδ T cells trigger rapid immune responses and are cytotoxic for porcine endothelial target cells. The strategies of *hFasL* transgene targeted at these innate-like T cells may reduce their cytotoxicity. **(D)** Neutrophil-mediated rejection. The *hCD31* transgene on porcine endothelial cells can suppress neutrophil-mediated xenogenic cytotoxicity *via* the inhibition of NETosis (histones, antibacterial peptides, and serine proteases). **(E)** DC-mediated rejection. Inflammatory cytokines-activated human DC is cytotoxic to porcine aortic endothelial cells by controlling human NK cell responses and pig CD8^+^ T lymphocytes. The hTRAIL transgene on pig DCs can decrease human T cell proliferation.

SIRP-α, a key inhibitory receptor on the surface of macrophages, binds to CD47, which effectively prevents phagocytosis (**Figure 2A**) (79). However, the cross-species incompatibility between porcine CD47 and human SIRP-α leads to the elimination of xenografts by host macrophages upon SIRP-α engagement (80). Remarkably, baboon macrophages phagocytose pig endothelial cells and podocytes in a similar manner as human macrophages. Zeng et al. generated the *GGTA1^-/-^*/*hCD47* transgenic Bama miniature pig to avoid hyperacute rejection and weaken the phagocytosis by host macrophages (81). In relation to lungs, the most difficult xenotransplantation, Watanabe et al. found that transgenic expression of hCD47 could mitigate diffuse hemorrhagic changes and antibody/complement deposition in pig-to-baboon lung xenotransplantation (21). The authors also published the first evidence of lung graft survival beyond 7 days (maximum survival to 10 days) in baboons. Moreover, Zhang et al. transplanted liver grafts from the *GGTA1^-/-^*/*hCD47* pig (Bama n = 3, Wuzhishan n = 3) to Tibetan macaques with the modified Sur II (HA-abdominal aorta + HV-inferior vena cava) procedure, and found that Tibetan macaques that had received liver xenografts exhibited a high level of inflammatory cytokine and leukocyte infiltration rather than severe coagulation disorders or immune rejection (survival to 14 days) (82). Nevertheless, further investigations on the function of hCD47 in other organ transplantations are needed because the function of macrophages varies by organ.

The CD200 binds to the CD200 receptor (CD200R) on macrophages, causing less secretion of pro-inflammatory cytokines and more secretion of anti-inflammatory cytokines (**Figure 2A**) (83). Sakai et al. observed that human CD200 expressed by swine endothelial cells suppressed xenogeneic rejection by CD200R^+^ human macrophages *in vitro* (84). *In vivo*, Yan et al. uncovered that hCD200 decreased human peri-graft macrophage infiltration and improved porcine xenograft survival in humanized mice to a greater extent than hCD47 (22). Furthermore, swine cells also express other inhibitory ligands, such as sialic acid-binding Ig-like lectins (Siglecs) (85), surfactant protein D (an oligometric C type lectin) (86), T-cell immunoglobulin and immunoreceptor tyrosine-based inhibitory motif domain (TIGIT) (87), HLA-G1 (an MHC Ib molecule) (88), and HLA class I histocompatibility antigen alpha chain E (HLA-E) (89), all capable of suppressing macrophage-mediated cytotoxicity and proinflammatory cytokine production. Although overexpression of human α-2,6-sialyltransferase (α2,6-ST) in swine endothelial cells has been found to prevent macrophage-mediated cytotoxicity *in vitro*, there is no *in vivo* evidence for the function of α2,6-ST (**Figure 2A**) (23).

## NK cells

NK cell infiltration was found in rejected xenografts beyond the hyperacute period, leading to adhesion and cytotoxicity in human NK cell-porcine endothelial cell interactions (90). Experiments *in vitro* demonstrated that human NK cells may induce pig endothelium lysis *via* two main immune mechanisms: antibody-dependent cytotoxicity (ADCC) and direct NK cell cytotoxicity. In the ADCC pathway (**Figure 2B**), natural and elicited antibodies in primate blood bind to α-Gal or non-Gal antigens on pig endothelium. The NK cells recognize and bind to the Fc region of these deposited antibodies through CD16 (an Fc receptor, also called FcγRIIIa), and then release granzyme- and perforin-containing cytotoxic granules that trigger pig endothelium apoptosis (91). The ADCC can be abolished by removing IgG *via* immune absorption and blocking with an anti-CD16 antibody (92). In the direct NK cells cytotoxicity pathway (**Figure 2B**), the downregulation of inhibitory signals and stimulation of activating signals result in the release of lytic granules. The major ligands recognized by inhibitory NK cell receptors are MHC class I (MHC-I) molecules (93). When the human NK cell receptor recognizes intraspecific MHC-I ligands (HLA class I, HLA-I), their toxicity would be inhibited (94). However, the MHC-I ligand (swine leukocyte antigen class I, SLA-I) on pig endothelium cannot effectively transmit inhibitory signals to the human NK cell, leading to their activation and the release of lytic granules (95). Moreover, the inhibitory receptors on human NK cells, such as KIR, ILT2, and CD94/NKG2A, also poorly recognize the SLA-I and pig HLA-E ortholog, consequently disabling inhibitory signals for NK cell activation (96). In addition, the upregulation of activating signals could also lead to direct NK cell cytotoxicity. Kim et al. tested a broad array of NK receptors including NKp46, 2B4, CD49d, CD48, CD2, and NKG2D, and found that only CD2 and NKG2D were involved in both cytotoxicity and cytokine (TNF-α and IFN-γ) production against porcine targets (97). The pig CD58 ortholog interacts with CD2 and interruption of this interaction through a monoclonal antibody inhibits lysis of porcine targets by human peripheral blood mononuclear cells (98). The porcine UL16-binding protein 1 (pULBP1) serves as a functional porcine ligand for human NKG2D to trigger human NK cell cytotoxicity (99). Thus, the modification of potential ligands on porcine tissues presents an attractive target to protect porcine xenografts from human NK cell cytotoxicity.

HLA-E, a heterodimer consisting of an α heavy chain and a light chain (β-2 microglobulin [B2M]), binds to the inhibitory receptor CD94/NKG2A of human NK cells. To inhibit direct NK cell cytotoxicity, Weiss et al. generated *hHLA-E*/*hB2M* transgenic pigs and found that pig endothelium derived from these animals was protected against human NK cytotoxicity and inhibited NK-secreted IFN-γ (24). Rao et al. inserted an *HLA-G1* transgene, encoding a non-classical MHC-I protein, to the porcine ROSA26 locus, and created *GGTA1^-/-^*/*hHLA-G1* pigs (25). In fibroblasts, transplantable organs, and islets, the positive expression of HLA-G1 plays a central role in immune suppression by lowering IFN-γ production *via* T cells and proliferation of CD4^+^ and CD8^+^ T cells, B cells, and NK cells (25). To reduce xenoantigen expression and thus reduce the recipient’s immune response, Fu et al. generated *GGTA1^-/-^*/*B2M^-/-^*/major histocompatibility complex class II transactivator (*CIITA^-/-^*) triple-knockout pigs, named GBC-21, by CRISPR/Cas technology and found that the resulting elimination of swine leukocyte antigen class I could effectively alleviate xenogeneic immune responses and prolong pig organ survival (26). Although there is still not enough *in vivo* evidence to fully support the relevance of these strategies to protect pig xenografts from human NK cell-mediated injury, the generation of HLA-E transgenic pigs is a promising approach.

Progress in xenotransplantation research relies on nonhuman primate models; therefore, differences in NK cell biology between humans and monkeys should be considered when analyzing immune responses. Human NK cells are characterized by the expression of the neuronal-cell adhesion molecule N-CAM (CD56) and the absence of CD3 (CD56^+^, CD3^−^) (100). Depending on CD56 expression level, human NK cells are divided into two subgroups, CD56^dim^ and CD56^bright^ (101). CD56^dim^ NK cells, accounting for more than 90% of NK cells, are mainly cytotoxic, expressing high levels of CD16 with stronger killing activity, while CD56^bright^ NK cells, producing a large spectrum of cytokines, mainly play an immunomodulatory role, with a high expression of the IL-2 receptor rather than CD16 (101). Moreover, baboon NK cells are IL-2-responsive and thus exert low spontaneous cytotoxicity against both human (leukemic cell line K562) and pig (endothelial cell line J2) target cells. They exhibit a CD3 (–)NKp46(+)CD8(dim)CD16(+/-) or CD3 (–)CD8(dim)CD16(bright) phenotype (102). Currently, NKp46 is a preferred NK cell marker, with evident expression in humans, numerous mouse strains (including BALB/c mice), and three common monkey species (*i.e.*, baboon, rhesus monkey, and cynomolgus) (103, 104). Furthermore, other receptors such as NKp30, NKp44, NKG2A, NKG2D, and KIR/CD158 were found on baboon NK cells, while KIR, CD94/NKG2A, and NKp80 were found on rhesus monkey NK cells (105, 106). In summary, these results can help to identify NK cells more precisely in nonhuman primates and to better use nonhuman primates as preclinical models for studying the role of NK cells in porcine xenograft rejection.

## Innate-like T cells

The γδ T cells (about 2% of total T cells) are not MHC-restricted and seem to be able to recognize native proteins rather than require peptides to be presented by MHC molecules on antigen-presenting cells (APCs) (107). Gago et al. studied the xenoreactivity of human γδ T cells against xenogenic porcine endothelial cells *in vitro* and found that 38.9% of human γδ T cells were cytotoxic towards porcine endothelial target cells, while porcine endothelial cells engineered to produce hFasL were less susceptible to lysis (**Figure 2C**) (27). Specific strategies targeted at these innate-like T cells may be important in controlling the innate cellular response to xenografts and facilitating graft survival.

## Neutrophils

The histopathological features of AHXR in a *GGTA1^-/-^* heart are similar to those seen in HAR (interstitial hemorrhage and edema), but include also a significant cellular infiltrate, mainly of polymorphonuclear neutrophils that account for 40–70% of white blood cells in humans (108). Neutrophils induce inflammation in xenografts through a unique form of cell death termed NETosis (**Figure 2D**) (109). Wang et al. unveiled that hCD31 on porcine endothelial cells suppressed neutrophil-mediated xenogenic cytotoxicity *via* the inhibition of NETosis *in vitro* (28). Although neutrophil-mediated immune rejection is not as strong as T lymphocyte-mediated immune rejection, neutrophil infiltration and aggregation can be found in all transgenic pigs, necessitating further research.

## Dendritic cells

Manna et al. substantiated that IL-15 activated human peripheral blood DCs and promoted the secretion of granzyme B from DCs, as well as observed that DCs are cytotoxic to porcine aortic endothelial cells (**Figure 2E**) (110). A human TNF-related apoptosis-inducing ligand (hTRAIL) transgene was inserted into a *GGTA1^-/-^*/*hCD46* transgenic pig, and it was observed that hTRAIL was mainly expressed in the spleen and lymphoid tissues in this *GGTA1^-/-^*/*hCD46*/*hTRAIL* transgenic pig (29). *In vitro*, hTRAIL-expressing porcine DCs were co-cultured with human PBMCs or isolated T cells, which demonstrated that transgenic DCs decreased human T cell proliferation, suggesting that they can possibly attenuate T cell responses against pig-to-primate xenografts (**Figure 2E**) (29). Human monocyte-derived DCs induced with IL-10 *in vitro* controlled human NK cell responses by reducing IFN-γ production, and inhibited cytotoxic CD8^+^ T lymphocytes (CTL) response against porcine endothelial cells (111). Because both DC subsets and maturation stages affect immune responses, further study should be executed on the complexity of DCs’ biology under the physiological and pathological conditions of xenotransplantation.

### Avoiding adaptive cellular xenograft rejection *via* genetic modifications

The APCs transport antigens from the xenotransplant to the peripheral lymph nodes, where they mediate initial T and B lymphocyte activation. The resulting T and B effector lymphocytes eventually migrate to the engraftment site and/or produce anti-graft high affinity antibodies, thereby enhancing immune rejection (112).

**Figure 3:**
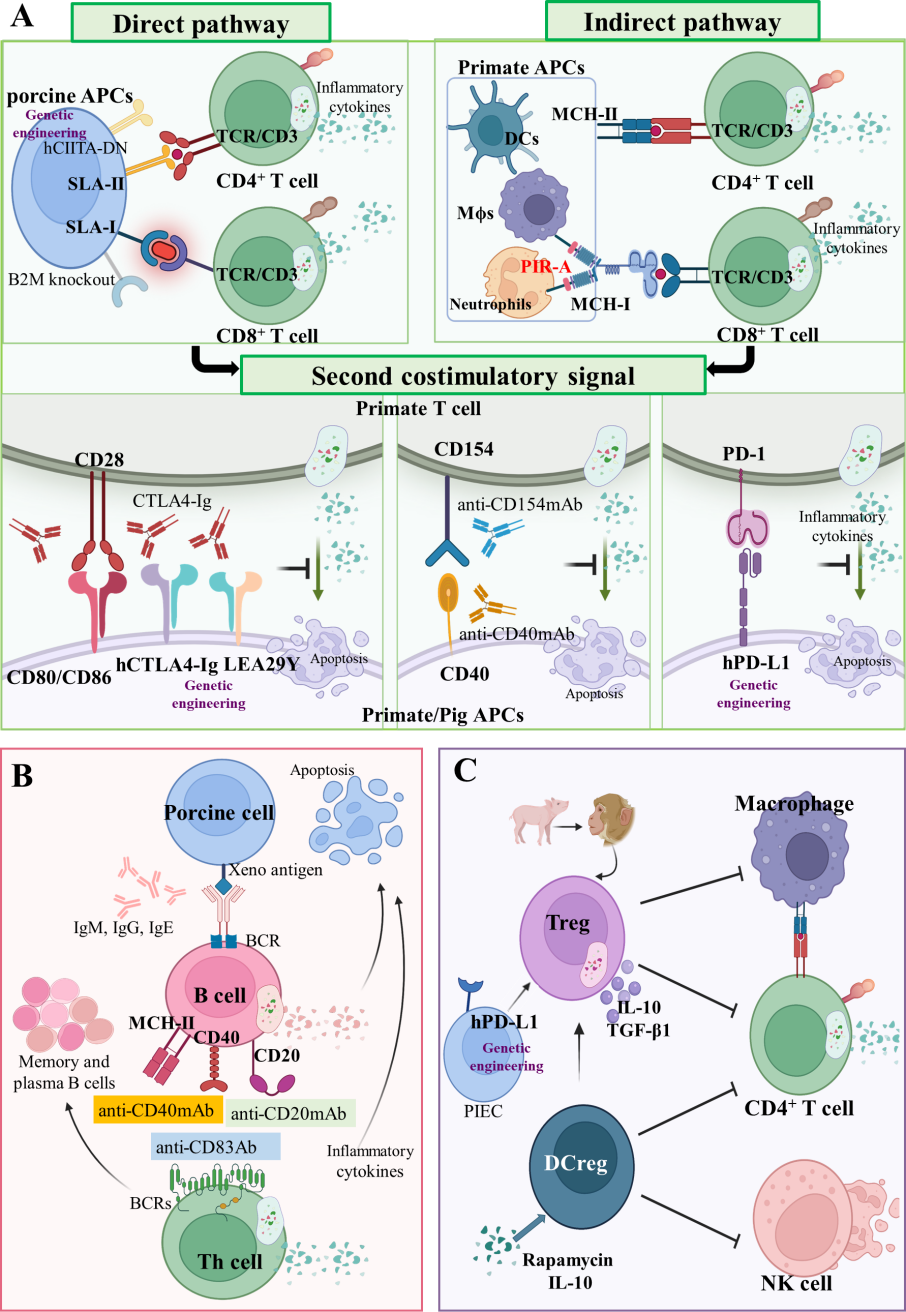
Adaptive cellular xenograft rejection **(A)** T cell response in xenograft rejection. In the direct pathway, T cell receptors (TCRs) of CD4^+^ and CD8^+^ T cells in primates interact with SLA-II- and SLA-I-peptide complexes on pig APCs, respectively. Human dominant-negative mutant class II transactivator (*CIITA-DN*) transgene and porcine beta-2-microglobulin knockout (*B2M^-/-^*) can reduce T cell direct immune response. In the indirect pathway, TCRs of CD4^+^ and CD8^+^ T cells in primates interact with MHC-II and MHC-I peptide complexes on primate APCs, respectively. The paired immunoglobulin-like receptor-A (PIR-A) monocytes and macrophages can also directly bind to MHC-I antigens to promote graft rejection. Three important costimulatory signaling axes have been identified in xenotransplantation, namely CD80/CD86-CD28, CD40-CD154 and PD-1/PD-L1 axis. Drugs targeting these costimulatory signals have been administrated, such as CTLA4-Ig, aCD154mAb, and aCD40mAb. Moreover, the hCTLA4-Ig, LEA29Y, and hPD-L1 transgenes can also inhibit these costimulatory signals. **(B)** B cells’ response in xenograft rejection. As antigen-presenting cells, B cells present antigen on MHC-II, CD40, or CD20 the receptor–ligand axis to helper T cells. Some drugs, such as anti-CD83 Ab, anti-CD20 mAb, and anti-CD40 mAb, can control B cells’ function and infiltration of inflammatory cells. **(C)** Regulatory immunological cells response in xenograft rejection. Regulatory T cells (Treg) and regulatory dendritic cells (DCreg) are immunological cells with a negative regulation function. The hPD-L1 transgenic on pig iliac endothelium cells (PIEC) can increase proliferation and suppressive potency of Tregs.

*T cells*. In xenotransplantation, recipients T cells can recognize graft antigens by two main distinct pathways: *via* the direct pathway as intact MHC xenoantigens on the surface of donor cells (pig APCs), and *via* the indirect pathway as self-restricted processed xenoantigens (pig-derived peptides on primate APCs) (113) (**Figure 3A**). A donor organ can only transport a finite number of passenger APCs, thus, the direct pathway’s significance in xenograft rejection reduces over time. Notably, the indirect pathway is available for antigen presentation for as long as the graft is in place, making it the long-term dominant mode of xenorecognition.

In the direct pathway, T cell receptors (TCRs) of CD4^+^ and CD8^+^ T cells in primates interact with SLA-II and SLA-I peptide complexes on pig APCs (porcine endothelial cells and passenger dendritic cells) (**Figure 3A**) (114). After constructing a human dominant-negative mutant class II transactivator (*CIITA-DN*) transgene, Hara et al. found that the expression of SLA-II on APCs from *CIITA-DN* pigs was significantly reduced (30). The absence of SLA-I in pigs was implemented by abrogating the porcine *B2M* gene, which negatively impacted the viability of the deficiency pigs (survival times < 4 weeks) (**Figure 3A**) (115). Recently, it was reported that PBMCs from *GGTA1^-/-^*/*B2M^-/-^*/*CIITA^-/-^* triple-knockout pigs were significantly less effective than wild-type in inducing human CD4^+^ and CD8^+^ T cell activation and proliferation (26). In fact, SLA molecules are involved in protective immune responses in pigs; therefore, inactivating them may decrease the human immune system’s ability to monitor transplanted pig organs for infectious disease. However, the elimination of SLA-II-contributed humoral xenoantigenicity can be carried out by modifying epitopes in SLA proteins (116).

In the indirect pathway, TCRs of CD4^+^ and CD8^+^ T cells in primates interact with MHC-II and MHC-I peptide complexes on primate APCs (**Figure 3A**) (37). Unlike the TCRs of CD8^+^ T cells, the paired immunoglobulin-like receptor-A (PIR-A) on monocytes and macrophages can directly bind to MHC-I antigens and promote graft rejection (**Figure 3A**) (117). In humans, PIR-A relates to other leucocyte immunoglobulin-like receptor family members (LILRs, also termed immunoglobulin-like transcripts, leucocyte immunoglobulin-like receptors, and monocyte/macrophage immunoglobulin-related receptors) (118). Recently, it was demonstrated for the first time that both macrophages and monocytes in mice lose their antigen-specific immune memory by blocking PIR-A with antibodies or by genetic deletion (117). This finding may shed light on chronic immune rejection, which has perplexed the xenotransplantation research community for more than 60 years.

The antigen-specific signals of xenotransplantation are delivered to the T cell through TCRs-CD3 and a second costimulatory signal (119). Amongst many costimulatory signals, we discuss the following three groups: (1) the B7 family (CD80/CD86) on APCs, binding the T cell costimulatory molecules CD28 (120); (2) The TNF/TNF receptor family, the prototype receptor–ligand pair of which is CD40–CD154 (121); (3) and the programmed cell death 1 (PD-1 or CD279) and programmed cell death ligand 1 (PD-L1 or CD274) receptor system (122) (**Figure 3A**). The CD80/CD86–CD28 axis can be blocked by the cytotoxic lymphocyte-associated molecule-4 (CTLA-4 or CD152) that has homology with the T cell antigen CD28 and serves as a ligand for CD80/CD86 (123). Treatment with soluble recombinant CTLA4-Ig was shown to extend islet graft survival after allotransplantation of pancreatic islet grafts in monkeys (124). Based on this result, Phelps et al. created *pCTLA4-Ig* and *GGTA1^-/-^*/p*CTLA4-Ig* transgenic pigs that exhibited robust expression of the pCTLA4-Ig protein in all organs and circulating in the blood (31). Vabres et al. transplanted the corneas from *hCTLA4-Ig* transgenic pigs to cynomolgus monkeys (*Macaca fascicularis*), and could establish that the expression of hCTLA4-Ig prolonged the final rejection time to 70 days (21 days in the wild-type group) (32). In addition, Bahr et al. established a genetically modified pig line with ubiquitous expression of LEA29Y, a human CTLA4-Ig derivate that binds human CD80/CD86 with high affinity. They found that LEA29Y expression blocked T cell co-stimulation without affecting sexual reproduction (33). The CD40–CD154 axis is effectively blocked by anti-CD154 mAb and anti-CD40 mAb, which prevents T cell response and extends the xenograft’s survival significantly (35, 125, 126). In the PD-1–PD-L1 axis, hPD-L1 transgenic pigs are characterized by the expression of hPD-L1 in the kidney, heart, and pancreas, as well as a reduced capacity to stimulate proliferation of human CD4^+^ T cells (34).

The treatment approaches for immunosuppressive regimens in xenotransplantation were initially derived from regiments used in allotransplantation, such as treatments with tacrolimus (FK506, FK) that blocks T cell cytokine production (54), cyclosporine (CsA), MMF, ATG/ALG that induce Fas-mediated T cell apoptosis (127), and corticosteroids (Cs). Moreover, the monoclonal antibodies (mAbs) introduced subsequently, such as CTLA-4Ig, anti-CD40 mAb, anti-CD4/8 mAb that depletes CD4^+^/CD8^+^ T cells (128), anti-CD154 mAb, and anti-CD20 mAb that depletes B cells (129), substantially improved the acceptance of xenografts. An alternative to reconstitute tolerized host T cells is the transplantation of the donor thymus in thymectomized and T cell-depleted immunocompetent host (130). The newly developing T cells are similarly exposed to both host and donor tissues and lead to loss of reactivity to both host and donor, inducing deletional T cell tolerance (131). Transplantation of porcine thymus as a composite “ thymokidney “ or a single vascular thymus lobe can support early primate thymopoiesis, which in turn induces T-cell tolerance to solid organ xenografts (18, 132). The approaches discussed above, combined with developments in immunosuppressive therapies, especially costimulatory blockers, have allowed long-term organ graft survival in NHPs.

*B cells*. It was demonstrated that costimulation blockade with an anti-CD40 antibody and anti-CD20 antibody prolonged the survival time of a heterotopic xenograft by controlling B cells function and infiltration of inflammatory cells (129, 133, 134). Wong et al. found that the anti-CD20 antibody rituximab depleted over 80% of recipient B cells, and further added the inhibitory effect of anti-CD83 Ab for CD83+ human dendritic cells and B cells They observed that anti-CD83 Ab only depleted activated (not resting) B cells and dendritic cells and therein reduced CD4^+^ T cell responses (135). In summary, B cells are essential in eliciting anti-non-Gal antibodies and targeting these cells allows the delay of xenograft rejection (**Figure 3B**).

*Regulatory immunological cells.* The use of host T regulatory cells (Tregs) contributes to the prevention or delay of xenograft rejection by controlling the activation and expansion of donor-reactive T cells (136). Herein, Ding et al. observed the significant proliferation of human CD4^+^/Foxp3^+^ Tregs and CD4^+^/Foxp3^-^ T effector cells in a co-culture system of pig iliac endothelium cells (PIECs) and human blood, and found that hPD-L1 transgenic PIECs inhibited Teff proliferation, while increasing the proliferation and suppressive potency of Tregs (**Figure 3C**) (137). Ezzelarab et al. observed that using regulatory dendritic cells (DCregs) in a model of allogeneic kidney transplantation these allowed a prolonged median graft survival up to 56 days, when compared with allotransplants receiving only costimulation blockade (CTLA4 Ig) and tapered rapamycin (median graft survival 39 days) (138). Madelon et al. differentiated human monocytes into DCregs *via* rapamycin or IL-10 *in vitro*, and uncovered that both rapamycin- and IL-10-induced DCregs caused significantly less IFN-γ production and human NK cell degranulation in response to porcine endothelial cells (**Figure 3C**) (111). Thus, DCregs can be a useful tools to promote xenograft tolerance.

## Coagulation dysfunction in pig-to-nonhuman primate transplantation

**Figure 4:**
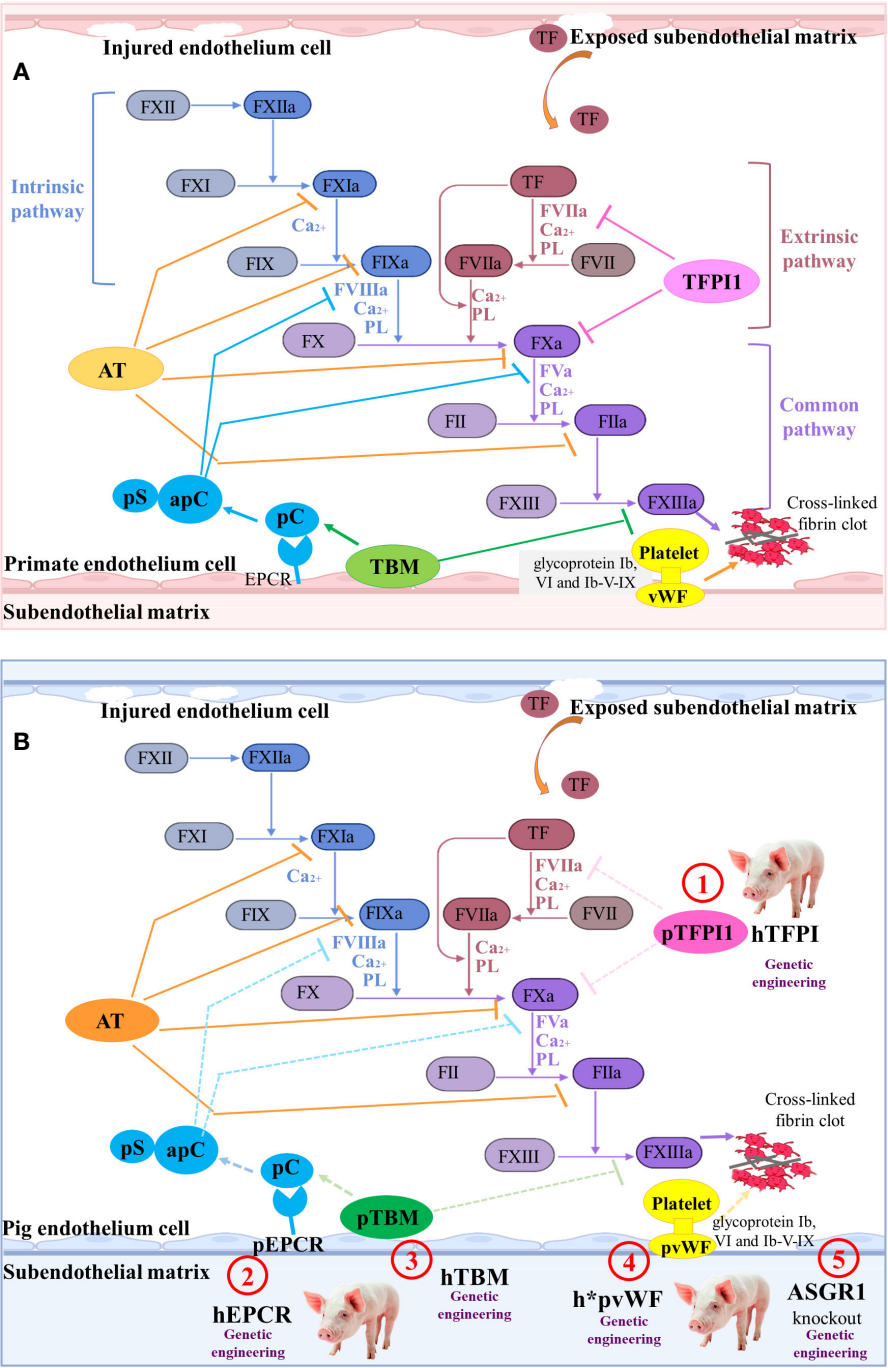
The coagulation cascade relating to pig-to-primate xenotransplantation **(A)** Coagulation cascade in primates. Coagulation factor VII (FVII) in the blood is the promoter of the intrinsic coagulation pathway. Damage to the blood vessel wall exposes the sub-endothelial cells expressing tissue factor (TF) to the blood stream, initiating the extrinsic coagulation pathway regulated by calcium ions. Additionally, in response to shear stress, von Willebrand factor (vWF) interacts with glycoprotein Ib (GPIb), VI, and Ib-V-IX on platelets, which results in platelet activation and adhesion. The clot formation is regulated by several anticoagulant molecules, such as tissue factor pathway inhibitor-1 (TFPI1), antithrombin (AT), Protein C (pC)/protein S (pS), and thrombomodulin (TBM). **(B)** Mechanisms of coagulation dysfunction after pig-to-primate xenotransplantation. The molecular incompatibilities between primate and pig coagulation–anticoagulation systems exaggerate the coagulation cascade. The pTFPI, pig endothelial protein C-receptor (pEPCR), pTBM, and pvWF are not sufficient to inhibit the blood coagulation factor of primates ineffectively shuts down coagulation (dashed lines indicate inadequate inhibition). Thus, the associated gene modification sites are identified as ① to ⑤.

When the HAR, AHXR, and T cell responses are prevented, coagulation dysregulation becomes more obvious following xenograft transplantation (46). Antibodies and complement cause the endothelial cells of the graft to change from an anticoagulant state to a procoagulant state, and induce immune cell infiltration and destruction of the blood vessel wall. Owing to the damage of the blood vessel wall, the sub-endothelial cells expressing tissue factor (TF) become exposed to the lumen of the blood vessels, thus initiating the extrinsic coagulation pathway regulated by calcium ions (139) (**Figure 4A**).

In order to overcome coagulation dysfunction, several coagulation-related pivotal factors, such as thrombomodulin (TBM), endothelial protein C-receptor (EPCR), tissue factor pathway inhibitor (TFPI), ectonucleoside triphosphate diphosphohydrolase-1 (CD39) that is an anti-thrombotic and cardiovascular protective mediator, and ecto-5’-nucleotidase (CD73), can be overexpressed to extend graft survival (140). Cardiac-specific expression of hCD39 in pigs can reduce myocardial dysfunction and the infarct size following ischemia-reperfusion injury (38). When *GGTA1^-/-^/hCD46*/*hTBM* was used as the donor pig and the pig heart was transplanted into the nonhuman primate model, the survival time was extended to 945 days (35). Although the survival time of the pig cardiac xenograft with six-gene modifications (*GGTA1^-/-^/hCD46*/*hCD55*/*hTBM*/*hCD39*/*hEPCR* or *GGTA1^-/-^/hCD46*/*hCD55*/*hEPCR*/*hCD47*/*hTFPI*) in the baboon was only 200 days, no significant coagulopathy or consumptive thrombocytopenia was observed in the six-transgene cohort (36). The recipient baboon succumbed to septic shock without consumptive coagulopathy or protein-losing nephropathy.

To control thrombocytopenia and consumptive coagulopathy even further, the pig von Willebrand factor (*VWF*) and asialoglycoprotein receptor 1 (*ASGR1*) genes can be edited (**Figure 4B**). The pig vWF activates primate platelets *via* their GPIb receptors, and this molecular incompatibility spontaneously aggregates primate platelets even without shear stress (141). Connolly et al. created the *GGTA1^-/-^*/*hCD46*/h*pVWF modified pig by replacing a pig *VWF* (*pVWF*) gene region-encoding glycoprotein Ib-binding site with human cDNA orthologs (39). After pig-to-baboon lung transplantation, organs from pigs with h*pVWF demonstrated reduced platelet sequestration during lung and liver perfusion *ex vivo* with human blood (39). Primate platelets were bound and phagocytosed by the ASGR1 on pig sinusoidal endothelial cells, which led to lethal thrombocytopenia in liver xenotransplantation (142). Livers from *ASGR1^-/-^* pigs exhibited decreased human platelet uptake both *in vivo* and *in vitro* (40, 41). Therefore, a graft from a specific genetically engineered pig and an effective immunosuppression has great potential to prevent immune injury and delay coagulation dysfunction.

## Systemic inflammation in xenotransplantation

The use of transgenic pigs expressing human heme oxygenase-1 (hHO-1) or human A20 (hA20) seems to be helpful in reducing systemic inflammation in xenograft recipients, because both hHO-1 and hA20 have anti-inflammatory and anti-apoptotic effects (42). Transgenic hA20 expression protects pig cells against human TNF-α-mediated apoptosis and partially against CD95(Fas)/CD95(Ligand) pathway-mediated cell death (143). The porcine kidney from *GGTA1^-/-^*/*hHO-1*/*hA20* transgenic pigs can successfully be perfused with diluted human AB-pooled blood for a maximum of 240 min (blood flow ceased after ∼60 min in wild-type kidneys) (42). A soluble human TNF-α receptor 1-IgG1 Fc chimeric protein (shTNFRI-Fc) could inhibit the binding of human TNF-α to pig TNF receptors and prevent human TNF-α-mediated inflammation and apoptosis, prompting researchers to generate *shTNFRI-Fc*/*hHO-1* and *CMAH^-/-^*/*GGTA1^-/-^*/*shTNFRI-Fc*/*hHO-1* transgenic pigs (43). In spite of the lack of *in vivo* xenotransplant data to confirm the superiority of these multigene-edited pigs, it is speculated that they are suitable for xenotransplantation owing to their ability to overcome hyperacute, acute, and anti-inflammatory rejection.

## Porcine endogenous retroviruses as a potential risk in xenotransplantation

Before the emergence of the CRISPR-Cas9 technology, the most appropriate method to reduce PERV expression was RNA interference. Transgenic pigs were produced by transfecting porcine fibroblasts with lentiviral vectors expressing the corresponding short hairpinRNA (144, 145). In addition, antiretroviral drugs, such as integrase inhibitors, have proven effective in inhibiting PERV infection *in vitro* (146). However, now that researchers have completely inactivated the pig endogenous retroviruses through CRISPR-Cas9 to produce pigs that cannot release infectious PERVs, antiretroviral drugs no longer seem necessary (44). Although it is still unclear whether PERVs can infect human, PERVs inactivated by CRISPR-Cas9 are currently the only way to ensure the inhibition of PERVs transmission during pig-to-human xenotransplantation (147).

## Recent clinical experiments using genetically modified pig organs

Montgomery et al. successfully performed two cases of GGTA1^-/-^ thymokidney in brain-dead human recipients, and given 1000 mg of methylprednisolone (daily) and 1000 mg of mycophenolate mofetil (intravenous, two times a day) until the kidney was explanted to induce immunosuppression. They observed no inflammation in renal tubules, arteries, glomeruli, capillaries in the subsequent 24 and 48 hours, but detected focal C4d deposition at 54 h in the xenograft for one recipient (148). Additionally, Porrett et al. published the results of a transplantation of bilateral kidneys from ten-gene modified pig (GGTA1^-/-^/CMAH^-/-^/β4GalNT2^-/-^/GHR^-/-^ (growth hormone receptor gene knockout)/hCD46/hCD55/hCD47/hTBM/hEPCR/hHO-1) into a brain-dead human recipient (149). The GHR^-/-^ is a reasonable approach to reduce the rate of growth and ultimate size of organ-source pigs, which is reviewed comprehensively by Iwase et al. (150). For the induction of immunosuppression, daily methylprednisolone taper, 6 mg/kg ATG and anti-CD20 antibodies were administered. Subsequently, mycophenolate mofetil, tacrolimus, and prednisone were given for the maintenance of immunosuppression for 74 h. Histological examination on day 1 and day 3 post-transplantation showed tubular injury with mild to extensive acute tubular necrosis, but no evidence of hyperacute rejection, cellular rejection, or deposition of antibody or complement proteins. These pig kidneys produced some urine, but did not process creatinine, suggesting that they weren’t functioning properly. Although this preclinical human model is useful to answer important logistical questions about the feasibility of xenotransplantation, the possible immune rejection and physiological dysfunction that would appear after several weeks of the transplant are not ascertainable.

On January 7, 2022, the first successful transplant of a heart from a 10-gene edited pig into a 57-year-old man with life-threatening heart disease, and life support was withdrawn on day 60 (151). For the depletion of B-cells, Rituximab was used and for the depletion of T cells, ATGs were infused pre-transplant. Complement C1 esterase inhibitor was also used to inhibit the complement. To inhibit CD40-CD154 mediated B cell activation and Type 1 immune responses, humanized monoclonal antibody KPL-404, which blocks CD40 co-stimulation, was administered. 1000 mg methylprednisolone was given on the day of xenotransplantation. To maintain immunosuppression, mycophenolate mofetil, KPL-404, and a rapid taper of methylprednisolone (from 125 mg daily to 30 mg daily) was administered. In contrast to a typical a transplant rejection, the final histology examination of the heart, which gained twice its initial weight, showed scattered myocyte necrosis, endothelial cell swelling, interstitial edema, and red-cell extravasation (151). Furthermore, infection with the porcine cytomegalovirus (pCMV) was detected starting on day 20 using plasma mcfDNA PCR testing. In nonhuman primate recipients, pCMV has been previously shown to be associated with shortened survival time of the transplant by increasing levels of inflammatory factors such as IL-6 and TNFα (152). It is obvious that highly sensitive detection methods combining PCR and serologic tests and strategies for the complete elimination of pCMV from donor herds are required.

## Conclusions

The current consensus regarding the initiation of clinical trials of xenotransplantation proposes that it is necessary to use genetically modified pigs with deletion of the major carbohydrate antigens reacting with the human natural antibodies, and with the addition of transgenes preventing complement and coagulation activation. Further specific transgenes modulating innate or adaptive immune responses, might be used depending on the organ or tissue transplanted. Slower types of rejection might be difficult to detect in these early human models because of the insufficient duration of the studies. Thus, a more thorough understanding of the immunological barriers, especially delayed innate/adaptive cellular xenograft rejection, will be the next groundbreaking process.

Regarding the immunosuppressive regimen, the blockade of the co-stimulatory pathway CD40-CD154 is essential to prevent the formation of new anti-pig antibodies. Initial trials with anti-CD154 mAb showed promising results in attenuating T cell response in pig to NHP models (153), yet further research showed that it had thrombogenic effects and has been discontinued in clinical use due to the presence of CD154 molecules on human platelets, or the IgG receptor FcγRIIA activation on platelets *via* immune complexes between anti-CD154 mAb and CD154 (154, 155). It is therefore necessary to use anti-CD40 mAb, as it has been shown to be equally effective in blocking the CD40-CD154 interaction (35). In recent trials of pig to human transplant, anti-CD40 mAb (KPL-404) was used as a part of the immunosuppressive regimen along with other immunosuppressants, including Rituximab (anti-CD20 mAb) and ATG, and had been preliminary shown to be safe for use in humans. Ultimately, optimizing genetically engineered organ-source pigs and advanced immunosuppressive regimen strategies potentially offer hope to patients with failing organs.

## Author contributions

LB, YW and CG-G developed the idea. TL, LC, and KW wrote the manuscript. TL and SD prepared figures. KW prepared the table. LB, CG-G and YW revised the manuscript. All authors contributed to the article and approved the submitted version.
